# Economic disadvantage and transitional outcomes: a study of young people from low-income families in Hong Kong

**DOI:** 10.1080/02673843.2014.928783

**Published:** 2014-08-08

**Authors:** Steven Sek Yum Ngai, Jacky Chau-Kiu Cheung, Siu-ming To, Hui Luan, Ruiling Zhao

**Affiliations:** ^a^ Department of Social Work, The Chinese University of Hong Kong, Shatin, New Territories, Hong Kong, China; ^b^ Department of Applied Social Studies, City University of Hong Kong, Hong Kong, China

**Keywords:** school-to-work transition, low-income families, extra-familial resources, resilience, youth, Hong Kong

## Abstract

This study draws on data from focus groups involving 50 young people from low-income families in Hong Kong to investigate their school-to-work experiences. In line with the ecological–developmental perspective, our results show that contextual influences, including lower levels of parental involvement and lack of opportunities for further education or skill development, constrain both the formulation and pursuit of educational and career goals. In contrast, service use and supportive interactions with parents and non-family adults were found to help young people find a career direction and foster more adaptive transition. Furthermore, our results indicate a striking difference in intrapersonal agency and coping styles between youths who were attending further education or engaged in jobs with career advancement opportunities and those who were not. We discuss the implications of our findings, both for future research and for policy development to enhance the school-to-work transition of economically disadvantaged young people.

## Introduction

Rapid social and economic changes in recent decades have dramatically altered young people's experiences of the transition from compulsory education to post-school life in many advanced industrial economies (Organization for Economic Cooperation and Development, 2010). Such transition now involves the negotiation of diverse pathways to various destinations including employment, further education or even disengagement from both work and education (te Reile, 2004). For young people to be adequately prepared for the school-to-work transition, intervention and support are required during the compulsory school years to develop occupational knowledge, social competence and career planning skills (Creed, Muller, & Patton, 2003). Most young people have access to such intervention and support through consistent home and school environments in which parents and school personnel provide social, emotional, practical and financial assistance. However, the development of further education and work aspirations for a significant number of young people, especially those living in low-income families, is more complex.

Economic disadvantage can force people to live under the great pressure of social exclusion (MacDonald & Marsh, 2002). For young people growing up in low-income families, their developmental environment is usually even more challenging, with inaccessible healthcare and welfare support, inadequate educational opportunities and resources, unavailable mentors and models within their social networks, and frequent exposure to antisocial peer groups and temptations from illegitimate opportunities (Billett et al., 2010). They are likely to remain idle because many of them have left school with little cultural capital to pursue study or work opportunities (Creed, 1999). It is vital that these young people receive help to break out from the traps of low-income environment, idleness and social exclusion. Moreover, without a job or an opportunity to study, these young people are at great risk of financial, social and eventually mental and behavioural problems (Schaufeli, 1997). At the extreme, problems such as criminal behaviour can be immensely traumatic to society and their financial and mental problems already inflict a sizeable social cost (Dixon, 2007). During the current era of financial fragility, the issue of youth transition under economic hardship is increasingly imperative and has already aroused much concern among researchers and policy-makers around the world (Johansson & Höjer, 2012).

In this study, we draw on research findings from eight focus group interviews with 50 young people living in low-income families in Hong Kong to investigate the transitional outcomes of this population and identify the barriers to and facilitators of their educational and career pathways. Disadvantaged young people exist everywhere and their problems are not confined to any particular culture. In Hong Kong, both economic disadvantage and youth transition have been the subject of public concern and academic research in recent decades. According to a study conducted by the Hong Kong Council of Social Service (2009), the number of young people aged 15–24 living in low-income families has increased to 20%, compared to 10% a decade ago. Moreover, as a result of the economic downturn since 2008, the unemployment rate for young people aged 15–24 has been hovering at around 15–20%, whereas the general population's unemployment rate during the same period was only around 4% (Census and Statistics Department, 2012). In addition, the number of both secondary and post-secondary students receiving financial assistance (i.e. fee remission assistance and means-tested grant and loan assistance) has risen dramatically in recent years (Student Financial Assistance Agency, 2012). Given this social trend of rising youth poverty, investigating youth transition and the barriers and facilitators involved provides us with insights into helping young people cope with economic disadvantage and achieve better developmental outcomes.

## Theoretical framework

Informed by an ecological–developmental perspective, we conceptualise transitional outcomes in relation to the goodness of fit between individual aspects (resources and developmental needs) and requests or opportunities offered by the multiple social contexts in which development occurs (Bronfenbrenner & Ceci, 1994; Ceci & Hembrooke, 2001). Accordingly, we distinguish among (1) outcomes of development characterised by an increase in personal resources through engagement in further education after compulsory schooling or a job that provides opportunities for career advancement and (2) outcomes of stagnation indicated by engagement in a job with limited potential for career advancement or disengagement from both work and education (Hendry & Kloep, 2002). From this perspective, we conceptualise engagement in further education or an adequate job with advancement opportunities as positive outcomes because they promote the further enrichment of skills and education, which is particularly important for economically disadvantaged young people (Polesel, 2010). However, the risk of social exclusion related to engagement in a job with limited career opportunities or disengagement from both work and education is inherent in bringing insufficient skills to the workplace, resulting in a lack of confidence in the potential for professional growth (Bonica & Sappa, 2010). These issues lead to stagnation and progressive deterioration, as they imply a reduction of personal and motivational resources in the face of the flexibility and ability to build new skills, which now more than ever characterise all professional contexts (Shanahan, 2000).

Moreover, according to the ecological–developmental perspective, whereas distal context factors such as a family's socio-economic status can identify the resources for or constraints on school-to-work transition, the actual mechanisms of development are more immediate proximal factors that involve the interpersonal interactions between the developing youth and other individuals across multiple developmental contexts (Bronfenbrenner & Ceci, 1994; Hendry & Kloep, 2002). Accordingly, we argue that proximal factors such as supportive interactions with parents, teachers, social workers and other extra-familial adults may function as protective mechanisms that positively influence the developmental trajectories of economically disadvantaged young people. At the family level, we explore whether parental emotional support and involvement in career planning facilitates more successful transitional outcomes (Alexander, Entwisle, & Kabbani, 2001; Schoon, 2006). At the community level, we study the extent to which these young people discuss their future school and work plans with non-family adults and whether they perceive those discussions as helpful. We assume that extra-familial help in transition planning such as vocational training, social work and other similar services are essential factors enhancing youth transition (Cauce, Stewart, Rodriguez, Cochran, & Ginzler, 2003; Croninger & Lee, 2001; Dufur, Parcel, & McKune, 2008). Previous research has identified some successful programmes and activities such as those that encompass transition planning services like job seeking, attending vocational training and counselling, and engagement in life-long learning (Cheung & Ngai, 2004; Ngai, Cheung, & Ngai, 2012; Phillips, 2010). Other studies, however, have been unable to agree on the effectiveness of some of these services (Esbensen & Osgood, 1999; Furlong, 2006). The exact service factors that are beneficial to economically disadvantaged youths need to be explored further before we can determine how they can be used by young people to enhance their transitional outcomes.

The ecological–developmental perspective also calls attention to the critical role played by the individual, a view similarly found in the research on resilience (Egeland, Carlson, & Sroufe, 1993; Masten et al., 1999). From this perspective, resilience is conceptualised as an ability to use both internal and external resources to resolve developmental issues at various life stages. Previous research has indicated that two resilient traits – a sense of agency and an instrumental coping style – are important proximal individual resources in the transition to adulthood (Sacker & Schoon, 2007). Hence, we suggest that young people with a sense of agency and an active instrumental coping style are more likely to use familial and extra-familial resources to achieve better transitional outcomes. Regarding young people's sense of agency, we investigate their educational motivation, future aspirations and ability to bring about desired outcomes through their efforts (Wayman, 2002). In relation to the instrumental coping style of young people, we explore their ability to accept help from others when needed and to elicit advice from parents, teachers, social workers and other non-family adults (Schoon, 2006).

Overall, we argue that young people's transitional outcomes are influenced by distal context factors (e.g. family socio-economic status) acting in concert with more immediate proximal factors (e.g. supportive interactions with parents, teachers, social workers or other extra-familial adults). In accordance with the resilience perspective, it is assumed that proximal resources can compensate for a higher-risk environment with fewer distal resources (e.g. living in a low-income family) to increase the likelihood of successful transition.

## Method

Focus group interviewing is used as a method of data collection because it is compatible with our research purpose, that is, an in-depth understanding of the transitional outcomes of economically disadvantaged youths to identify the barriers to and facilitators of the further education or work engagement of this group. In this study, economically disadvantaged youths refer to young people who grew up in a deprived home environment with a monthly family income at or below 75% of the median monthly domestic household income (MMDHI) (Social Welfare Department, 2010a; see Table 1). In Hong Kong, the government uses this criterion to assess which families are eligible to receive fee waivers for social services (Social Welfare Department, 2010b). Accordingly, in spring of 2012, we conducted eight focus group interviews with 50 young people who fulfilled the aforementioned criterion to investigate their views and experiences regarding their school-to-work transition. We specifically targeted young people aged 19–24 because this is the critical age range in the transition to young adulthood – a time characterised by important choices about employment or higher study (Dixon, 2007). The interviewees were identified through non-governmental organisations delivering community-based youth centre service or outreaching youth social work service in Hong Kong. Informed consent was obtained after we explained the purpose of the study and the procedure to be followed. There was no obligation to participate in the study and an assurance of confidentiality was given in a cover letter before recruitment. The research design and the methods used in this study were assessed and approved by an ethical review committee from the Chinese University of Hong Kong.

**Table 1:** Median monthly domestic household income (MMDHI) by household size 2009.

Household size	MMDHI	75% of MMDHI
1	HK$6500 (US$833)	HK$4875 (US$625)
2	HK$13,500 (US$1731)	HK$10,125 (US$1298)
3	HK$18,000 (US$2308)	HK$13,500 (US$1731)
4	HK$22,300 (US$2859)	HK$16,725 (US$2144)
5	HK$29,000 (US$3718)	HK$21,750 (US$2788)
6	HK$32,000 (US$4103)	HK$24,000 (US$3077)
7 and over	HK$37,000 (US$4744)	HK$27,750 (US$3558)

An interview guide, in terms of general questions, was used to keep the narrative within this study's frame of reference. The first section of the interview guide was designed to elicit information on transitional outcomes and included questions on the school and job experiences of the participants at the time of the interview. Questions about school status indicated whether the participants were enrolled in a post-secondary education programme and whether they had completed or were working to complete the programme. We asked employed interviewees about the quality of their job in terms of its link to future career opportunities. We asked unemployed interviewees about the frequency and duration of their unemployment and whether they had sought employment in the 12 months before the interview. The second section of the interview guide was designed to collect information on the barriers to and facilitators of young people's school-to-work transition. We explored what the interviewees had done to enhance their school-to-work transition and how they viewed their ability to bring about desired outcomes through their efforts. Moreover, we asked whether they had talked with or elicited advice from parents, teachers, social workers or other non-family adults. For those who had received social services, we asked what their experiences had been and whether they had found the services useful. We also examined what forms of interaction had occurred within the family realm and what their effects had been in addition to asking how they had benefited from familial and ex-familial help and support in school or work planning. A non-directive approach was used to allow the interviewees maximum freedom to articulate their experiences (Huberman & Miles, 2002; Padgett, 2008). There was no strict order of questioning and occasional prompts were used, if necessary, to facilitate the flow of the narration. Summarisation was used to provide the interviewees with feedback about what they seemed to be expressing and to check understanding. Each interview lasted about two hours and all were audio-taped and subsequently transcribed from Cantonese to English.

Our analysis of the interview transcripts was carried out in steps as follows (Burr, 1995; Huberman & Miles, 2002). First, we identified key terms and themes. Second, we examined these terms and themes with reference to the global organising features of the transcripts: major events, the protagonists and their relationships, and the network of terms around which the arguments were articulated. At the end of this process, we organised the material in accordance with the transitional outcomes and ecological–developmental factors described in our theoretical framework. Member checking was used to ensure that the analysis represented the interviewees' experiences as accurately as possible. According to Padgett (2008), this procedure requires researchers to verify their interpretations by going back to their research participants. This is an important step in guarding against researcher bias, as it demonstrates to participants that their views are not only taken into account, but are also valued as authoritative. In line with this procedure, the participants were invited to read our accounts of the interviews and to react to them, thus allowing emerging interpretations to be evaluated and refined. The participants' suggestions concerning transitional outcomes and the related barriers and facilitators were incorporated into the final analysis.

## Results

### Participant profile and transitional outcomes

The characteristics of our sample are shown in Table 2. The average age of the participants was 22 years (ranging from 19 to 24 years), with an equal proportion in terms of gender. Their educational attainment ranged from primary to university educations, among which 84% had secondary (grade 12) or less educational attainment and 16% had post-secondary (i.e. higher diploma, associate's degree or bachelor's degree) educational attainment. All of the participants lived in low-income home environments with a monthly family income at or below 75% of the MMDHI and parents working in manual labour jobs or jobless. The average household size among the participants was around four people, with 64% living in families with married parents, 26% coming from families with divorced parents and 10% from families with one or both parents deceased. Among the participants, 42% were receiving public assistance and 70% had received social services including community-based youth centres' career planning services and government training schemes for young school leavers.

**Table 2:** Participant characteristics (*N* = 50).

Characteristic	%
Gender	
Female	50.0
Male	50.0
Educational attainment	
Primary	2.0
Secondary (grades 7–12)	82.0
Post-secondary (higher diploma, associate's degree or bachelor's degree)	16.0
Occupational status	
Student	12.0
Employee	68.0
Unemployed	20.0
Parental marital status	
Married	64.0
Divorced	26.0
One parent/both parents deceased	10.0
Paternal educational attainment	
Primary	40.0
Secondary	52.0
Post-secondary	8.0
Maternal educational attainment	
Primary	44.0
Secondary	50.0
Post-secondary	6.0
Paternal employment status	
Employee	80.0
Unemployed	16.0
Homemaker	4.0
Maternal employment status	
Employee	54.0
Unemployed	4.0
Homemaker	42.0
Monthly family income	
HK$4875 (US$625) or below	20.0
HK$4876–10,125 (US$626–1298)	42.0
HK$10,126–13,500 (US$1299–1731)	16.0
HK$13,501–16,725 (US$1732–2144)	10.0
HK$16,726–21,750 (US$2145–2788)	6.0
HK$21,751–24,000 (US$2789–3077)	2.0
HK$24,001–27,750 (US$3078–3558)	2.0
HK$27,751 (US$3559) or above	2.0
Reception of community-based youth centres' career planning services or	
government training schemes for young school leavers	
Yes	70.0
No	30.0
Reception of public assistance	
Yes	42.0
No	58.0
Characteristic	Mean
Age (years)	21.7
Household size (number of persons/household)	4.1

At the time of the interviews, 12% of the participants were students, 68% were employed and 20% were unemployed. Using the transitional outcome classification scheme discussed in our theoretical framework, the participants were coded as having (1) outcomes of development if they were pursuing further education (i.e. post-secondary education) after compulsory schooling or engaging in a job that provided opportunities for career advancement, and (2) outcomes of stagnation if they were engaging in a job that provided limited potential for career advancement or were disengaged from both work and education (Bronfenbrenner & Ceci, 1994; Hendry & Kloep, 2002). Table 3 shows the distribution of the 50 participants across different transitional outcomes.

**Table 3:** Transitional outcomes of youth participants (*N* = 50).

Transitional outcomes	*n*	%
Outcomes of development		
Attending post-secondary education	6	12.0
Engaging in a job with opportunities for career advancement	6	12.0
Outcomes of stagnation		
Engaging in a job with limited potential for career advancement	28	56.0
Disengaged from both work and education	10	20.0

Note: Outcomes of development are defined by engagement in further education (i.e. post-secondary education) after compulsory schooling or a job that provides opportunities for career advancement. Outcomes of stagnation are defined by engagement in a job with limited potential for career advancement or disengagement from both work and education.

Although the majority of the participants had managed to enter the working world, a critical situation emerged due to the low percentage of students. We conceptualised engaging in post-secondary education as a positive outcome, as it promoted the further enrichment of skills and education (Polesel, 2010). Nevertheless, only 12% of the participants were attending post-secondary education. Moreover, only 12% of the young people who found work were employed through fixed-term contracts in the form of trainees or junior staff members in the fields of social welfare, education, fashion design, business and maintenance services. Together with the participants attending post-secondary education, they constituted the group with transitional outcomes of development (hereafter referred to as the TOD group). In contrast, a significant proportion of the participants (56%) had been hired into what they reported as inadequate jobs characterised by low-pay and low-skill work. Getting this type of job did not normally require young people to have specific qualifications, as employment depended on personal recommendations from peers or local adults. However, once employed in these jobs, young people soon realised that they had limited career prospects. Among the participants in inadequate jobs, 43% had previously had one to four brief work experiences and 57% had had at least five such experiences. Such a high job turnover may be an expression of an inability to adapt to the work context; however, this condition may be justified by conscious choices to quit work perceived as unsatisfactory (Bonica & Sappa, 2010). Unemployment referred to the condition in which young people had not found a job in the course of the year despite actively seeking employment (20%). This population is probably at the greatest risk for social exclusion. The participants with inadequate jobs and those who were unemployed constituted the group with transitional outcomes of stagnation (hereafter referred to as the TOS group).

### Interactions with parents

All of the interviewees acknowledged that growing up in a low-income family did have consequences for their school-to-work transition. They noted that compared with their better-off peers, family poverty had constrained their learning resources (e.g. tutoring, books and computer aids) and created barriers to their educational and career pathways. Moreover, they did not see parents as a major source of advice about further education and work futures, particularly when the parents had limited educational backgrounds and thus were less able to promote a broad range of options for the interviewees.

Nevertheless, regarding interactions with parents, the TOD group reported a much greater frequency and intensity of connection to their parents than did the TOS group. The young people in the TOD group particularly valued the emotional support and career role modelling their parents provided. Emotional support included messages of encouragement such as ‘Never give up’ and ‘You can do everything if you work hard’. Some of the TOD interviewees also remarked that although their parents were less competent in assisting with education or employment, their parents always tried their best to provide a safe and stable home environment so that they could have the peace of mind to tackle challenges in their school-to-work transition.

Jenifer: The pressure of the university entrance examination is huge, so it's crucial to have the support and concern from parents. Apart from this, parental support serves as a keystone of my emotional wellbeing, while others aren't able to do the same. My parents will prepare a delicious meal and I can feel their love from this. They will also ask my sisters to quiet down, so I can do my revision.

The TOD group also reported obtaining practical support from their parents, including the latter monitoring school progress and exploring job or training programme opportunities. They consistently described parents serving as career role models who exhibited perseverance in the face of economic difficulties and demanding working conditions. Having family role models that were connected to the working world seemed to contribute to a greater sense of agency on the part of the TOD youths.

Ming: My mother has tried to help me as much as possible. Say if there's any shop in the mall that is recruiting, she would ask and see if I'm interested. With her encouragement and support, I'll go give it a try.

In contrast, young people in the TOS group reported that there was little discussion about school and future work plans with their parents because many of their parents were either unemployed or underemployed and, hence, preoccupied with a difficult family financial situation. Even in cases where the parents of the TOS interviewees were employed, the wages were so low that the parents often had to take more than one job to provide basic necessities for the family. As such, the TOS group expressed less emotional intensity or affect when discussing interactions with parents. Winnie, an unemployed youth in the TOS group, told us that her parents showed little concern about her studies, even when she started skipping school. She left school without completing her compulsory education and then switched from one low-skill job to another, ultimately becoming disengaged from both education and work.

Winnie: My parents don't have much time to pay attention to me, as they leave home before I wake up, and are not back before I sleep. Because of that, I have little communication with my parents. They wouldn't urge me to find a job. Now I live on their limited income and stay at home all the time.

According to the interviewees' narratives, poor family financial situations place many barriers to young people's educational and career pathways. Some of the interviewees ended up leaving school at an early age to take jobs with limited potential for career advancement, or they disengaged from work and education entirely. In addition to practical support, parents' emotional support affects young people's development. However, compared with the TOD interviewees, those in the TOS group received less guidance and support from their parents (in education or work), which in turn reinforced their disadvantaged life situations and negatively influenced their development.

### Sense of agency and instrumental coping

The interviewees' sense of self and individual coping styles also distinguished the TOD and TOS groups in this study. In particular, we found more frequent expression of feelings of low energy, often marked by a defeated, depressive state, in the narratives of the TOS group. Moreover, this group of young people had a passive stance in the face of difficulties and their higher prevalence of passivity and low energy appeared to be an immobilising force both in compulsory schooling and in the years since dropping out. Even if they had some job goals, they tended to be much less clear about what was required to enter a particular job field. Lack of life and career planning was very evident, with most of the TOS young people expressing a lot of uncertainty about the steps they needed to take to achieve their goals and how they could support themselves and live independently.

Fai: After doing many odd jobs, none of the jobs I have done is ideal. I still don't see the goal of my life. I feel like I'm just floating in the middle of the ocean.

Daisy: I feel useless because I didn't study hard at school. Now I am 21 years old but I know almost nothing. I regret that very much. Talking about the future, I don't know what to say.

In contrast, the TOD group expressed higher energy both while in compulsory schooling and during the transition period afterward. This group of young people was also more likely to adopt an active rather than a passive stance in the face of challenges. This active stance fuelled their ability to seek out new pathways to adult roles, as exemplified in the following narrative. Kevin, a TOD interviewee, decided to pursue an aircraft maintenance engineering programme at a vocational training institute even though he was qualified for a university education. Kevin quickly found a job as an apprentice in an aircraft maintenance company after graduation from the programme. The satisfaction related in his narrative stemmed from the expression and recognition of competence that developed during his vocational training and was then highly recognised by his employer.

Kevin: When I started working in this company I really knew a lot. The foreman was surprised too. He said, ‘You attended a really good school.’ In fact I can see it too. Now, after one year, I feel like I'm in control of my life.

Kevin's trajectory is an example of what Hendry and Kloep (2002) define as transitional outcomes of development. The reinforcement of a competent self initiated by the vocational training experience resulted in further development and enrichment during the work experience (Polesel, 2010; Schoon, 2006). Such development also extended to the external context, leading Kevin to invest further in his education by attending an advanced aircraft maintenance engineering programme provided at a polytechnic university.

Moreover, the TOD group more often displayed an active instrumental coping style by accepting help from others when needed and by crediting others as sources of support. In contrast, the TOS group was less trusting of others and had a harder time talking with or eliciting advice from teachers or social workers. Such a difference in interactions with extra-familial adults is discussed in the following section.

### Interactions with extra-familial adults

#### School setting

School is an important developmental context because, apart from the dissemination of knowledge, there are other aspects of education that contribute to the development of young people, such as the care and support of teachers and connections with community resources (Alexander et al., 2001; Dufur et al., 2008). This kind of support is especially important for young people coming from poor households, which often means not having access to information and resources about further education and work futures (Croninger & Lee, 2001). Most of the young people in the TOS group reported that they did not get much assistance from the school system. Schools were depicted as stressful places in which they were not able to concentrate on their studies or manage rules and regulations. Although a teacher's role is to assist students with educational and personal development needs, most of the TOS interviewees who did not do well in their studies felt that teachers were insensitive to the learning needs of disadvantaged students and unwilling to help them catch up and start anew.

Peter: When we went to the teachers for help, they tended to think that we couldn't understand the reference materials anyway, so why would they even bother to teach?

Some of the TOS interviewees also reported unstable educational experiences due to frequent changes in family residence during childhood, which created a sense of marginalisation and exclusion from the school system.

Ying: When I was small, my family moved frequently, as a result I have studied in many schools. By that time, all I was thinking was that I would have to move to a new place anyway, so bonding with people was not worth it. I therefore did not manage to establish relationships with my classmates and teachers.

In contrast, the TOD group reported a higher level of connection to specific teachers or school programmes due to their higher school motivation and better academic performance, which allowed them to be easily identified by teachers and thus receive extra attention and assistance. Indeed, almost all of the TOD interviewees indicated at least one teacher in school who was very important. The following narrative from Christine, a TOD interviewee who had completed her university education and was working as a human service professional, shows how teacher support can make a difference in the lives of disadvantaged youths by building their self-esteem and providing access to community resources.

Christine: During my secondary education, my academic results were very outstanding. Since my teachers knew about my family's financial condition, whenever chances for applications of financial aid became available, they would encourage me to apply for the aid. They would also assist me, guide me and give me a lot of advice throughout the application process. At that time, I felt I was being admired and recognized. This made me want to continue to do well or even better.

The interviewees' narratives show that while school has the potential to provide useful support for young people from low-income households, the availability of such support varied among the interviewees in this study. Compared with the TOD group, the TOS group reported receiving less support from (and thus less connection to) the school system. Moreover, our research suggests that teachers appeared to pay less attention to disadvantaged students who did poorly in their studies. The risk factors associated with economic disadvantage, including the lack of stability in their upbringing, have also created barriers for these young people to establish interpersonal ties at school, which limits their opportunities for successful transition.

#### Community settings

Interactions with extra-familial adults also distinguished the TOD and TOS groups in our study. Specifically, young people in the TOS group were quite isolated from extra-familial realms and less able to elicit or recognise support in their communities. Many of them did not have a relationship with an adult in the community on whom they could rely, and they saw themselves as very much on their own in making their way in the world. In contrast, young people in the TOD group described more connections with other adults within their current education or work roles and emphasised the importance of community programme involvement. Some of the successful programmes include community-based youth centres' career planning services and government training schemes for young school leavers.

The career planning services mentioned by the TOD group were conducted at least twice weekly by non-governmental organisations with drop-in sessions at community-based youth centres (Cheung & Ngai, 2004; Ngai et al., 2012). The provision included light refreshments, recreational facilities and support from social workers offering advice on education and employment. Conversations usually took place around the snack bar where a range of topics were discussed. However, if a young person wanted to talk with a social worker on a one-to-one basis, they could move to a quieter corner of the centre. The majority of young people in the TOD group reported having participated in this kind of career planning service. They remarked that such services had created a safe space for them to make new friends and develop mutual support. In addition, the social workers provided valuable academic and career guidance, which enhanced their social competence and life reflection.

Tak: The influence was really big. My social worker understood our impoverished economic situation and cared about our difficulties in education and work. He helped look for information on education and employment opportunities, discussed them with us, and gave us advice. In fact, after we met the social worker, we now have a mutual support group and we feel like we belong to it. The more we know, the more we think, thus, we've developed new ways to handle difficulties and challenges.

The above narrative shows how the TOD interviewees valued practitioners who were helpful, kind, gave good advice and listened in an understanding manner. These positive experiences illustrate a mentoring relationship between disadvantaged youths and supportive non-family adults who provide opportunities for social and emotional development (Cauce et al., 2003; Friesen & Brennan, 2005). An important aspect of this mentoring relationship is the way in which it becomes embedded in reciprocal forms of mutual support fuelled by the increased self-understanding and enhanced self-esteem of disadvantaged youths (Phillips, 2010). These aspects of self can strengthen inner resources, enabling young people to tackle different life challenges and move forward in their school-to-work transition.

The government training schemes highlighted by our interviewees involved the provision of a wide range of pre-employment training such as job search and interpersonal skills, computer operation and vocational skills for young school leavers. Career guidance is available whereby case managers assist the participants to map out career plans according to their interests and abilities. Moreover, these schemes provide on-the-job training in the form of internships that last 6–12 months. As an incentive for more enterprises to engage in the schemes, a monthly training subsidy is offered to participating employers who appoint an experienced staff member to mentor the youth participant (Labor Department, 2013).

However, our research shows that the TOD and TOS groups differed in how they interpreted the benefits they received from the government training schemes. In particular, most of the young people in the TOS group did not learn many useful skills through the schemes and still felt confused about their futures. Some TOS interviewees joined the programmes just to kill time or avoid the nagging of their parents. They also reported a number of problems pertaining to the ways in which the schemes were run. Not surprisingly, many of the young people in the TOS group responded by either skipping attendance or dropping out altogether.

Ling: The so-called ‘courses’ mostly aim at teaching very basic things, and those who are enrolled in the class only want to have fun. I went for it because my mother was nagging me about jobs all the time. I used this as an excuse so that she wouldn't bother me anymore.

Fai: The case managers of the governmental training schemes wouldn't inspire us to think about our future. However, they are supposed to lead us to think about where we want to go. As we're already floating helplessly on the ocean, how could we possibly figure out our paths ourselves?

In contrast, the government training schemes were regarded as quite useful among the TOD interviewees who had set specific career goals and knew what they wanted to do. They reportedly treasured and appreciated the guidance and support from their mentors during on-the-job training, as reflected in the transitional experience of Tony, who left school early but regained self-confidence and was employed as a fashion design trainee after joining the government training scheme.

Tony had a keen interest in fashion design and regarded it as his career goal. After dropping out of school, he attended a governmental training scheme, took several short courses on fashion design and eventually secured an internship opportunity in a fashion design company. Tony acknowledged the important role played by his mentor, an experienced staff member at the host company, who provided extensive teaching, supervision and encouragement during on-the-job training. The internship enabled Tony to move from a context of adversity and disengagement to one characterised by support in learning career-relevant skills. Aspects of the on-the-job training that contributed to his transitional outcome of development are described in the following excerpt.

Tony: My mentor treated me with respect, praised me for my accomplishments, honestly criticized my work when it was inadequate, challenged me with gradually more technically demanding tasks and with greater responsibility. We worked shoulder to shoulder when I experienced difficulty, with my mentor patiently explaining, demonstrating and expecting me to rise to the challenge.

Tony's case illustrates a key factor of resilient school-to-work transition – the beneficial interactions with a supportive adult in the extra-familial realm (Croninger & Lee, 2001; Bonica & Sappa, 2010). Although Tony described the support he experienced from his mentor, who was proud of his accomplishments, he remained humble about all he still had to learn and eager to continue learning. Due to his high level of commitment and competence in fashion design, Tony was able to secure a trainee contract in the host company. Tony's mentor became his role model, inspiring Tony to enrol in a diploma programme provided at a vocational training institute to pursue fashion design as a career goal.

## Conclusions

In this study, we draw on research findings from eight focus group interviews with 50 young people from low-income families in Hong Kong to investigate their transitional outcomes and identify the related barriers to and facilitators of further education or work engagement. Based on an ecological–developmental perspective, we distinguish among (1) outcomes of development (TOD) characterised by an increase in personal resources through engagement in further education after compulsory schooling or a job that provides opportunities for career advancement, and (2) outcomes of stagnation (TOS) indicated by engagement in a job with limited career advancement potential or disengagement from both work and education (Bronfenbrenner & Ceci, 1994; Hendry & Kloep, 2002). We also examine extra-familial, familial and individual resources to determine what might help young people to establish an educational or career pathway subsequent to dropping out of school or completing compulsory education.

Our findings indicate that the majority of these young people had TOS status, whereas only a small proportion had TOD status. Among the TOS interviewees, those who had left school early were at the greatest risk for social exclusion, as most of them were unemployed and had not found a job over the course of the year despite actively seeking employment. The results echo previous research on the developmental pathways of young people in poverty, showing that this population is among the most disadvantaged in school-to-work transition (Creed, 1999; Dixon, 2007).

The outcomes of our interviews with the TOS group are consistent with the ecological–developmental perspective (Bronfenbrenner & Ceci, 1994; Hendry & Kloep, 2002) in which contextual influences, whether personal (e.g. having a history of learning difficulties), familial (e.g. limited learning resources in a poor household and lower levels of parental acceptance and involvement) or extra-familial (e.g. lack of opportunities for further education or skill development and limited support from adults around school or the workplace), can constrain both the formulation and pursuit of educational and career goals. The TOS interviewees perceived themselves as facing barriers related to the instability in their upbringing, lack of adult interest in their life and few supportive relationships, indicating that they construed their circumstances as a potential barrier to later educational and occupational achievement (Billett et al., 2010; MacDonald & Marsh, 2002).

Meanwhile, our findings from interviews with the TOD group are in line with the ecological–developmental perspective that among economically disadvantaged youths, service use, supportive interactions with parents, teachers, social workers or other extra-familial adults can help young people find a career direction and foster more adaptive transitional outcomes. These conclusions reinforce previous findings that parental emotional support and involvement in career planning and extra-familial help in transition planning may function as protective mechanisms that positively influence the developmental trajectories of youths from poor households (Alexander et al., 2001; Cauce et al., 2003; Croninger & Lee, 2001; Ngai et al., 2012; Phillips, 2010; Schoon, 2006). The results of this study thus indicate that when disadvantaged young people obtain access to adequate resources and opportunities, they can evade failure and social exclusion. Furthermore, our study extends the literature on economically disadvantaged youths (Bynner & Parsons, 2002) by highlighting the need to go further in examining the factors that help this population resist the adverse effects of family poverty.

Our results also indicate a striking difference in intrapersonal agency and coping styles between the TOS and TOD groups. In particular, the concept of self-reliance was evident among the TOS group, who felt that there is no one else to rely on. Although such a self-perception can prompt achievements that exceed others' expectations, it can also be negative if young people strive to do well yet do not succeed, as they then have no one but themselves to blame. As previous research on resilient traits (Egeland et al., 1993; Masten et al., 1999; Schoon, 2006) suggests, resilience – a capacity that develops over time in the context of person–environment interactions – is linked to the individual's ability to use both internal and external resources to navigate developmental challenges. We find that the TOD group had a more active instrumental coping style than the TOS group, which is central to fostering resilience and enhancing school-to-work transition. Furthermore, our research suggests that a lack of interaction, support and engagement within the home, school and community settings of the TOS group led to considerable isolation from community resources. This isolation perpetuated a decreased sense of agency and contributed to a sense of hopelessness and passivity among the TOS group (Creed, 1999; Dixon, 2007). In the long run, this may lead to increased difficulty in finding successful educational or career pathways in young adulthood.

### Practical implications

Given the aforementioned findings, interventions based on the ecological–developmental perspective represent particularly appropriate ways of preventing further TOS status among economically disadvantaged young people. These types of interventions focus on bolstering resources and skills in addition to improving interactions between youths and adults across multiple developmental contexts (Cauce et al., 2003; Ngai et al., 2012; Schoon, 2006).

First, it must be acknowledged that young people from low-income families often face difficulties in becoming responsible and self-motivated, a developmental task that is surely more challenging for them than it is for their peers, given their disadvantages (MacDonald & Marsh, 2002). As such, economically disadvantaged youths need help during their school-to-work transition. They need to be exposed to diverse experiences and people so they can begin to form interests and develop future aspirations. They also need help with naming their skills and talents and matching these to possible education and work choices. Likewise, they need to be encouraged so that they develop the confidence to set goals and make sound choices (Creed et al., 2003). Due to their poor family financial situations, these young people should also have access to learning resources including tutoring, reference materials and computer aids (Ngai et al., 2012). These career development activities should start in the compulsory school years before important decisions must be made about further education or work engagement (Bonica & Sappa, 2010).

Moreover, young people in poverty circumstances need the support of institutions to improve their chances of successful transition. This pertains to educational institutions such as schools along with institutions offering career planning services such as community-based youth centres, where disadvantaged youths can interact with their peers and receive help from non-family adults such as teachers and social workers (Phillips, 2010). Such institutions are particularly important for young people from low-income households who typically do not have access to information and resources on educational and work opportunities. In this regard, our results show that by providing opportunities for psychosocial development and mutual support, the career planning services provided at community-based youth centres have enabled disadvantaged youths to achieve adaptive school-to-work transition. Regarding the important roles played by educational institutions, our research suggests that school reform in Hong Kong is necessary. According to the TOS interviewees who did poorly in their studies, most teachers appeared to be insensitive to the learning needs of disadvantaged students and were unwilling to help them improve. There are two remedies to this situation: resources and training for teachers, or the introduction of professionals with expertise into the school system (e.g. social workers from community-based youth centres). For instance, schools could develop career resources aimed at young people from low-income households and ensure that the teacher role encompasses a focus on future goals while also making the expertise of career development professionals available on a consultancy basis (Cauce et al., 2003). At the school policy level, targeting school stability, encouraging educational attainment and raising expectations about further education and work outcomes would be positive steps (Friesen & Brennan, 2005). Our interviews with the TOD group indicate that disadvantaged youths are most likely to display their strengths within such arrangements. Nevertheless, while there were young people in this study who did receive appropriate teacher guidance, it was not consistent and there was no evidence of any concerted attention paid to this aspect of development.

In addition, although we focus on youth poverty in particular, it is just one aspect of family poverty (Dufur et al., 2008). To foster more adaptive transitional outcomes for economically disadvantaged young people, integrated support must be organised. This includes providing adequate financial aid and effective support services for the family. In this connection, our research indicates that despite their best efforts to create a safe and stable home environment for healthy development of their children, many low-income parents are already overwhelmed by poverty that constrains their ability to support their children's school-to-work transition. Social welfare and related policies therefore need to establish poverty-sensitive qualifications for human service professionals and generate a social equity perspective among policy-makers and concerned stakeholders (Dominelli, 2004). This social equity perspective can be instrumental in working on the constraints faced by disadvantaged youths and their families, by seeking to provide targeted support (e.g. a poverty-resistant living subsidy and sustained mentoring by non-family adults) aimed at equalising differences in life chances between these young people and their better-off peers (Rahn & Chassé, 2009). In so doing, positive school-to-work transition experiences are possible through the creation of support for disadvantaged youths and a simultaneous effort to improve the basic conditions of family life.

Furthermore, our study pinpoints the vital importance of targeted vocational and employment support services for economically disadvantaged young people. Our interviews with the TOD group reveal an essential element of these services – the on-the-job training approach inspired by learning by doing and work-based learning (Polesel, 2010). The close link with local companies that on-the-job training provides also makes it possible to plan a learning experience that is more closely related to the actual demands of the working world (Creed et al., 2003). Another element of success in vocational and employment support services is the focus on emotional dynamics and relationships between youth participants and trainers or mentors (Bonica & Sappa, 2010), whose protective role in promoting the achievement and motivation of disadvantaged youths was treasured and appreciated by the TOD group in our study. Vocational and employment support services, at least among our sample of disadvantaged youths, also seemed to support a reconciliation with studying that leads many who have left school early to plan their scholastic reintegration (Shanahan, 2000).

In terms of operational implications, our findings suggest a pressing need to reform the existing government training schemes in Hong Kong by improving the quality of training programmes and strengthening case managers' support so that the schemes can be really beneficial to disadvantaged youths (Ngai & Ngai, 2007). Furthermore, promoting even more institutional incentives in order to encourage employers to support learning in the workplace is vital (Bonica & Sappa, 2010). However, fostering such reconciliation between learning and working requires a cultural transformation to overcome the perception that school and work are incompatible. As many commentators have remarked (e.g. Leney & Green, 2005), the idea of promoting youth transition seems impossible if employers' objective is to recruit young people who are available for low-pay jobs with limited career opportunities and at the same time competent yet submissive. Similarly, it is not possible to improve young people's relationships with the working world without offering concrete opportunities for flexible reintegration into educational or career pathways (Nurmi, Salmela-Aro, & Koivisto, 2002). Such issues appear particularly important among economically disadvantaged youths, who are perhaps also less ready to confront the working world autonomously. For them, it is even more important to guarantee additional spaces for learning and an accompaniment to job placement or successive schooling (Polesel, 2010).

### Research implications

This study is subject to several limitations that have implications for future research. First, our results were derived from a small sample of young people from low-income families in Hong Kong whose transitional processes and outcomes may differ from those of disadvantaged youths elsewhere. Hence, to increase the generalisability of the results, it would be helpful if comparative studies engaged similar groups of young people living in different settings and contexts. Moreover, given the cross-sectional research design, our classification scheme on the transitional outcomes of economically disadvantaged young people was necessarily crude, as it was developed to capture the status of this population at one moment in time (Hendry & Kloep, 2002). It overlooked much of the complexity of experience subsequent to dropping out of school or completion of compulsory education and was taken as a snapshot of youth transition during a time of considerable flux (Bonica & Sappa, 2010; Nurmi et al., 2002; Shanahan, 2000). Thus, in terms of further research, researchers addressing the question of economically disadvantaged youths' school-to-work transition should incorporate a longitudinal study of their trajectories, further exploring the processes through which these young people navigate different educational and employment pathways activated during the various micro-transitions of which they are the protagonists.

Despite these limitations, this study provides a fuller understanding of the transitional experiences of young people from low-income families, clarifying how extra-familial, familial and individual resources may help or impede these young people in pursuing further education or engaging in occupational life. Increased knowledge of the processes by which economically disadvantaged youths are prepared for the transition to young adulthood, how they think about their educational or employment pathways and the roles of various stakeholders can inform policy and service development to better meet the developmental needs of this population.
